# Real-time automatic hospital-wide surveillance of nosocomial infections and outbreaks in a large Chinese tertiary hospital

**DOI:** 10.1186/1472-6947-14-9

**Published:** 2014-01-29

**Authors:** Mingmei Du, Yubin Xing, Jijiang Suo, Bowei Liu, Na Jia, Rui Huo, Chunping Chen, Yunxi Liu

**Affiliations:** 1 Department of Infection Management and Disease Control, Chinese PLA General Hospital, 28 Fuxing Road, Haidian District, Beijing 100853, China; 2 State Key Laboratory of Pathogen and Biosecurity, Beijing Institute of Microbiology and Epidemiology, Beijing, China; 3 XingLin Information Technology Company, ZheJiang Province, HangZhou, China

## Abstract

**Background:**

We aimed to develop a real-time nosocomial infection surveillance system (RT-NISS) to monitor all nosocomial infections (NIs) and outbreaks in a Chinese comprehensive hospital to better prevent and control NIs.

**Methods:**

The screening algorithm used in RT-NISS included microbiological reports, antibiotic usage, serological and molecular testing, imaging reports, and fever history. The system could, in real-time, identify new NIs, record data, and produce time-series reports to align NI cases.

**Results:**

Compared with a manual survey of NIs (the gold standard), the sensitivity and specificity of RT-NISS was 98.8% (84/85) and 93.0% (827/889), with time-saving efficiencies of about 200 times. RT-NISS obtained the highest hospital-wide monthly NI rate of 2.62%, while physician and medical record reviews reported rates of 1.52% and 2.35% respectively. It took about two hours for one infection control practitioner (ICP) to deal with 70 new suspicious NI cases; there were 3,500 inpatients each day in the study hospital. The system could also provide various updated data (i.e. the daily NI rate, surgical site infection (SSI) rate) for each ward, or the entire hospital. Within 3 years of implementing RT-NISS, the ICPs monitored and successfully controlled about 30 NI clusters and 4 outbreaks at the study hospital.

**Conclusions:**

Just like the “ICPs’ eyes”, RT-NISS was an essential and efficient tool for the day-to-day monitoring of all NIs and outbreak within the hospital; a task that would not have been accomplished through manual process.

## Background

Surveillance of NIs is fundamental for NI prevention and control. It is essential to establish real-time surveillance systems and to undertake corresponding control measurements, to significantly decrease NIs and prevent outbreaks. The prospective, targeted surveillance of surgical wounds has prevented and controlled NIs for many years [1]. However, targeted surveillance can be time-consuming and only monitors the infections in a few selected wards, rather than covering an entire hospital. The National Nosocomial Infections Surveillance system (NNIS) revealed that only 20% of NIs occur in intensive care units (ICU) [2] and 19% were related to SSI [3]. These data suggest that hospital-wide surveillance should be enforced to detect all NIs [4]. Further, many NIs and outbreaks were not monitored and were out of control. The best way to manage NIs is to perform targeted and hospital-wide surveillance simultaneously. However, because the majority of hosptials have insufficient numbers of ICPs, an automated computer-based surveillance system should provide much needed asssistance.

Fortunately, several targeted surveillance computer programs have been developed in recent years [5,6]. These have assisted ICPs to good effect, and in some cases replaced manual surveillance having detected NIs in targeted wards, such as NIs associated with Catheter-Associated Bloodstream Infections (CABSI) [7], SSIs [8] and ICUs [9].

Most hospitals in China have large bed numbers, but few ICPs. This has made real-time hosptial-wide surveillance difficult to implement. Further, some special customs within Chinese hospitals (i.e. low rates of pathogen cultivation and high levels of antibiotic misuse) has made computer algorithm development more difficult. Currently, retrospective review of medical records and/or physician reports is usually performed to carry out hospital-wide surveillance. However, this type of manual surveillance is often time-consuming and the data can be inaccurate and incomplete.

We have developed a system, named RT-NISS, that is capable of daily, automatic, and real-time screening of all suspicious NIs cases and outbreaks. The system supports ICPs in large Chinese tertiary hospitals to undertake daily hospital-wide and targeted surveillance simultaneously, and with greater efficiency.

## Methods

This study was carried out in a tertiary hospital in Beijing with approximately 3,500 beds (including 201 beds in 11 ICUs); it performs 270 surgical operations each day, accepts 12,000 inpatients each month, with an average length of stay of 10 days. The hospital has the most advanced computer information system in China, including integrated hospital information system (IHIS), laboratory information system (LIS), imageology achieving system (RIS), and an anesthesia operation system (AOS).

The system uses data mining J2EE (Java 2 Platform, Enterprise Edition), AJAX (Asynchronous JavaScript and XML), and Flex computer technologies. It is operated with Oracle 10.0 database, Tomcat 6.0. The system automatically starts downloading data from the hospital information systems (e.g. IHIS, LIS, RIS, AOS) at 2.00 am, but data can also be downloaded manually at any time. The daily data can be collected and analyzed within 10 to 20 minutes.

For the purposes of managing inpatient data for this study, approval was obtained from the Medical Ethics Committee of the Chinese PLA General Hospital (approval number 11KMM51).

### NI definitions

NIs were defined according to the Chinese NI diagnosis criterion published by the Ministry of Public Health in 2001 [10], which differs somewhat from the Centers for Disease Control and Prevention (1988) [11]. First, in China, the definition for respiratory tract infection (RTI) includes the CDC categories of ‘pneumonia’, ‘lower respiratory tract infection other than pneumonia’, and ‘upper tract respiratory infection’ (if the inpatient has a fever of ≥38C for more than 2 days and a sore throat). Second, patients ≤12 months are included in the general population rather than as a separate group.

NIs were classified as RTI, urinary tract infection (UTI), bloodstream infection (BSI), SSI, gastrointestinal tract infection (GTI), skin and soft tissue infection (STI) and other infections, including parotitis, chickenpox, and neurological infections. An infection was considered to be a NI if the patient was diagnosed ≥48 hours after admission, and had no evidence of subclinical infection at the time of admission. NIs were diagnosed based on clinical diagnosis and pathogens diagnosis, that is, the NIs were in accordance with clinical diagnosis definitions, but they did not necessarily need the pathogen/s identified, i.e. for upper tract respiratory infection cases.

### RT-NISS NIs computer algorithm (Professional Suspicious NIs Screening Criteria)

The professional suspicious NIs screening strategy was based upon specific diagnostic criteria for certain infection sites; these took into consideration positive microbiological examinations, antibiotic administration, serological and molecular testing (e.g. C-reactive protein, calcitonin), positive imaging reports, temperature, invasive device use, and inpatient transfer data. Some screening strategies could be used for a variety of wards, but specific ones were needed for special wards. For example, the screening strategy for general SSI included: 1) continual fever (temperature ≥38.0C) for 2 days after surgery, but excluding a temperature within 72 hours after surgery (surgical fevers mostly occur during this time period); 2) positive microbiological examinations of surgical wound secretions; and 3) new antibiotic administration, excluding prophylactic use of antibiotics. Aside from these three conditions, the screening strategy for central nerve SSI after brain surgery included: 1) white blood cell (WBC) abnormality in cerebrospinal fluid (CSF); 2) antibiotic administration into the vertebral canal; and 3) increased demand for CSF testing post-surgery. All of the NIs algorithms were automatically performed on each inpatient; the system then alerted staff if the inpatients met any of the screening criteria.

### RT-NISS real-time NIs prewarning alerts

The NI computer algorithm could prewarn staff of all suspicious NIs, but it could not warn about new NIs inpatients every day. In order to realize real-time prewarning, the system was able to record NI-related information and undertake time-serial alignments of these cases. On the next day, when the system had detected new suspicious NIs, it could automatically use information collected on the previous day (as baseline) to avoid repeat alerting; new suspicious NIs were alerted, but the previously confirmed NIs were not alerted again. For example, a similar positive microbiological examination from a patient did not trigger the system again when the patient had already been recognized as having a NI. But with regards to a previously excluded alert, if the infection conditions worsened and met the standard criteria again, a new alert was triggered. For example, an alert of “fever for 3 days” for an inpatient was excluded; if the patient’s temperature increased the next day (i.e. “fever for 4 days”), or his/her infectious condition met other screening criteria (such as positive microbiological examination), then an alert would be triggered for this patient on the same day. Otherwise, there would be no subsequent alert for this inpatient under such conditions.

### RT-NISS NIs confirmation from prewarning alerts

**Figure 1:**
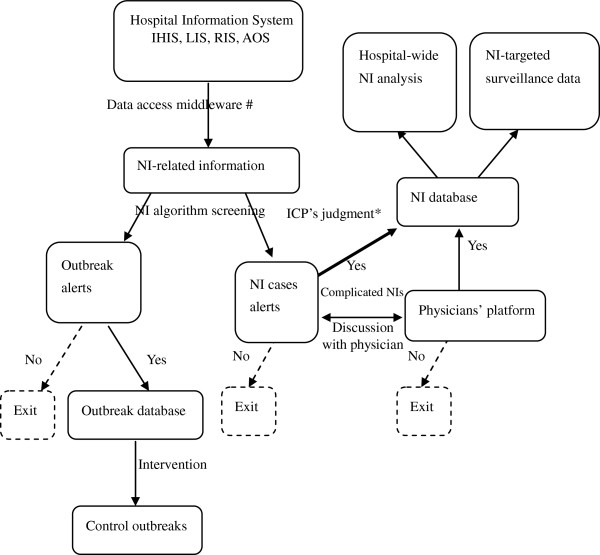
**A flow chart of NI cases and outbreak prewarning and confirmation.** *ICP’s judgment: the NI prewarning alerts could be confirmed by ICPs or physicians. The experience of the study hospital showed that it was better to depend on ICPs judgment (consulting some complicated NIs with physicians) because the physicians’ compliance was not good and some NIs were lost or delayed. The precision of ICPs confirmation is very important; it determined the accuracy of the relative NI rate. #Data Access Middleware: a kind of computer software that provides services to software applications from diverse data sources (IHIS, LIS, RIS, AOS); it makes it easier for RT-NISS to perform communication and input/output processes.

Each morning the ICPs were able to deal efficiently with all of the suspicious alerts with the support of RT-NISS. Most of suspicious alerts were confirmed by ICPs, but some complicated or questionable ones were confirmed after discussion with the physicians through the “interactive communication platform” between RT-NISS and the physicians’ workstation (such as via mobile telephone messaging). Further, the physicians could communicate with the ICPs about NI-related questions. A flow chart of NI prewarning and confirmation is illustrated in Figure 1. All of the confirmed NIs were calculated to create an overall prevalence for the wider hospital. Some special cases (ie. SSI, CABSI, multi-drug resistant pathogen infection) were arranged into a special targeted module to enable the ICPs to pay closer attention to these cases, and conduct relevant clinical interventions.

### RT-NISS NIs outbreak prewarning

Clusters and outbreaks were defined by the Chinese Ministry of Public Health in 2009 [12]. An NI ‘outbreak’ is identified as ≥3 NI cases with the same pathogenic infection (identified homologous) that occur in a single department during a short period (usually one week). The definition for ‘cluster’ is ≥3 cases with the same pathogenic infection detected in one department (usually within one week), or if the NI incidence of one department is higher than baseline levels when epidemiological surveys or homologous identification cannot be performed. The RT-NISS selected the prewarning threshold according to the type of cluster. The prewarning criteria included three parts: the same multi-drug resistant pathogen cluster (≥2 or ≥3 identical pathogens within 7 days on a single ward, according to the surveillance data history), diarrhea cluster (≥2 confirmed NIs with diarrhea within 7 days on one ward, or ≥3 patients with positive fecal testing within 3 days on one ward, excluding routine fecal testing at the time of admission, because the number of routine fecal tests would increase in special wards for viral diarrhea, which is indicative of a suspicious diarrhea outbreak), and SSI cluster (≥2 confirmed SSI inpatients within 7 to 21 days on one ward, according to the baseline SSI rates for that ward). In order to realize sensitivity around prewarning of outbreaks, the prewarning criteria combined NIs with community infections, and the microbiological reports included confirmed infections, colonization, or contamination; we considered that community infection is sometimes the source of outbreaks under certain circumstances, and the cross-spread of pathogens may cause infection, colonization or contamination in the hospital setting.

If a ward triggered the prewarning criteria, the system would highlight the name of the ward in red. The operator could obtain more detailed information about this alert by opening the file on the computer to access the inpatients’ ID, inpatients’ bed, infection pathogen, infected site, infection duration, admission time, attending physician, and such like. The ICPs were able to make preliminary judgments about the alert, as either dangerous or a false positive, and whether clinical investigations or interventions were required immediately for dangerous alerts.

## Results

### ICPs daily work with the help of RT-NISS

**Figure 2:**
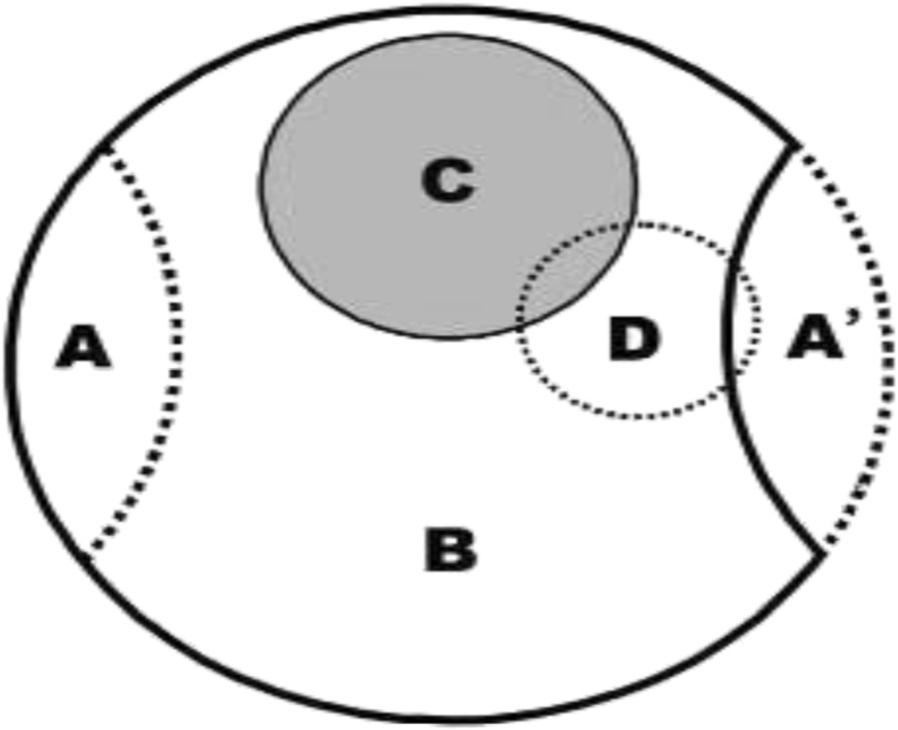
**The daily confirmed NIs inpatients and non-NI inpatients were kept in a dynamic balance. A**: daily new inpatients; **B**: non-NI inpatients; **C**: confirmed NI inpatients; **D**: daily new NI prewarning alert inpatients; **A**’: daily newly discharged inpatients. All inpatients were composed of **A**, **B** and **C**, without **A**’. **D** comes from **A**’, **B** and **C**. Most prewarning inpatients were confirmed in the **D** group; the others were excluded because they had community acquired infections, colonizations, or had not acquired a NI.

Each day at 2:00 am, the RT-NISS was activated, providing new NI alerts. At 8:00 am, the ICPs would diagnose approximately 70 prewarning NI alerts across our hospital. It would take 2 hours for the ICPs to confirm new NIs and to understand all of the NIs (including new and previously confirmed NIs) with their detailed information (i.e. infection site, ward, infection pathogen). Then, the ICPs would undertake interventions, where necessary, according to this data. In fact, the number of confirmed NIs cases was kept in a dynamic balance, between the daily new confirmed NIs cases, and some of the confirmed NIs cases that had been discharged or cured (Figure 2).

**Figure 3:**
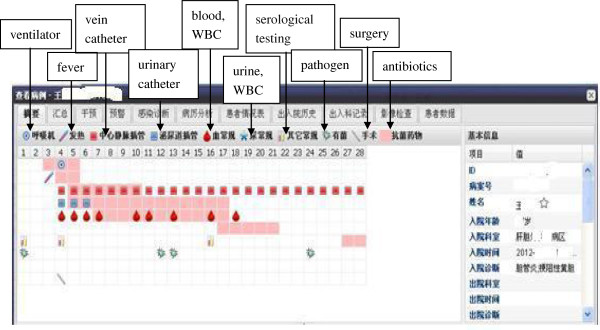
**Visual time-series chart of an inpatient including infection-related data with positive results.** The inpatients’ data are displayed using different colors, which enable the ICPs to make immediate decisions e.g. fever onset day, pathological examination results, the category and duration for antibiotic usage, use of invasive devices.

It would take 1 to 2 minutes for the ICPs to diagnose a suspicious NI with the help of RT-NISS. The RT-NISS provided many functions to assist the ICPs to make timely and accurate decisions. First, the system would provide a visual time-series chart of each inpatient with positive results concerning their infection-related information (Figure 3). Second, the RT-NISS would automatically connect to the medical records, and would use computer crawler technology to highlight the NIs key words with different colors (such as wheezy phlegm, frequent micturition, urgent micturition), which helped the ICPs to check the medical records more easily and quickly. Third, if one inpatient had the same test several times, for example WBC count, the RT-NISS would provide a graph to display increasing or decreasing values to guide the ICPs’ decision making.

### The accuracy and efficiency of RT-NISS

#### A comparison between RT-NISS and manual survey

In March 2013, we performed a manual survey (the “gold standard”) on NI rates to compare with RT-NISS findings. Because some of the ICPs were absent, we only manually-checked 974 inpatients from 29 wards (there were 3,498 inpatients on 102 wards at the time); this ensured more accurate data. The 29 wards belonged to the following four groups: 12 medical wards (i.e. respiratory, gastroenterology, cardiology, endocrinology, nephrology, oncology, hematology, and neurology), 11 surgical wards (i.e. general surgery, cardiac and vascular surgery, neurosurgery, urology, ophthalmology and otorhinolaryngology, orthopedics, and liver and gallbladder), 1 obstetrics and gynecology, 1 pediatric ward, and 4 ICUs. The manual survey was conducted by two physicians and one ICP for each ward (a total of 58 physicians and 5 ICPs). One ICP was in charge of five or six teams. All of the physicians and ICPs received six hours of training, and all of the team members had rehearsed the survey protocol for 2 days prior to the survey.

During the manual survey, which lasted up to two days, the team members visited inpatients and reviewed their medical records on the wards. The ICP of each team would supervise and control the data quality. The time cost for the manual survey was at least 756 hours (or 12 hours for each team member). However, it took only 3.5 hours for one ICP using RT-NISS to diagnosis 146 prewarning suspicious NIs from 974 inpatients. The time-saving ratio of RT-NISS was around 200 times over the manual survey.

Table 1 illustrates comparisons between the RT-NISS algorithm, RT-NISS plus ICP diagnosis, and manual survey of NI rates. RT-NISS prewarned 146 suspicious NIs, with a sensitivity of 98.8% (84/85) and specificity of 93.0% (827/889). The ICP diagnosed 80 cases from 146 alerts with a sensitivity of 94.1% (80/85) and specificity of 99.3% (883/889; Table 1). Overall, the agreement between the RT-NISS plus ICP diagnosis and the “gold standard” was favourable (kappa (k), 0.93).

**Table 1:** Comparison of NI detection through RT-NISS algorithm, RT-NISS algorithm followed by ICPs diagnosis, manual survey

	**RT-NISS algorithm**		**RT-NISS algorithm followed by ICP diagnosis**	
**Th**	**NIs**	**Non-NI**	**NIs**	**Non-NI**
**Manual survey:**				
**NIs**	84	1*	80	5**
**Non-NI**	62	827	6	883

*Case lost by RT-NISS algorithm because the case was diagnosed as mild urinary system infection (2 days slight muddy urine and fever for 1 day); there was no urine culture or antibiotics use.

**Among 5 cases, one case was lost by RT-NISS algorithm*; the other 4 cases were RT-NISS algorithm prewarned NI alerts, but were not confirmed by the ICPs because the clinical information provided by RT-NISS was insufficient.

### The comparison of monthly NIs rate between RT-NISS and other traditional reports

We compared three methods to calculate NIs rates of 10,765 in patients who were discharged in July 2011. The methods used were RT-NISS algorithm plus ICPs diagnosis, ICP manual review, and physician reports. All of the NI cases diagnosed by these methods were peer-reviewed by senior physicians. The RT-NISS obtained the highest prevalence with 2.62% (282/10,765), medical records review calculated a prevalence of 2.35% (253/10,765), and the physicians reported the lowest rate of 1.52% (164/10,765). The RT-NISS prewarned 948 suspicious alerts and confirmed 282 cases by the ICPs, so the negative-predictive value was 91.19% (9817/10,765). In summary, the system successfully reduced the number of patients the ICPs needed to manually review by surveillance by around 10 times.

### RT-NISS NI clusters or outbreaks alerts

There were about 50 alerts of NI clusters each year (one alert per week), and out of these about 10 serious alerts required clinical investigations and interventions. The other 40 false positive alerts may have been colonization, or community infections, and were excluded by the ICPs. Between 2010 and 2013, the ICPs who used RT-NISS successfully controlled the majority of serious NI clusters (about 30 clusters) because of the early prewarning and their immediate and effective control measures. Only four outbreaks occurred and all were controlled within a short time; one involved a SSI, one norovirus diarrhea, and the other two were concerned with multi-drug resistant pathogen infections. However, before 2010 the ICPs only recognized one severe multi-drug resistant pathogen outbreak using manual surveillance. Most of the clusters or outbreaks would have not been detected via surveillance, without applying RT-NISS.

### RT-NISS statistical analysis function and targeted surveillance

**Figure 4:**
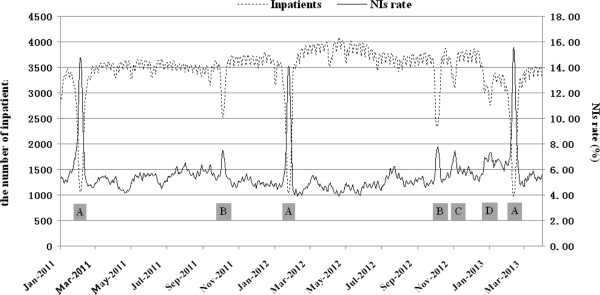
**Daily NI rate in the wide hospital.** The daily NI rate was stable at about 4.0 to 6.0%. The NI rate and the number of inpatients were kept in range. The NIs rate exceeded 6.0% during special period A, B, C and D. The main reason was the number of inpatients was less than usual during these periods. Most inpatients were relatively severe because less acute inpatients did not see a doctor, or were not admitted. A: the Spring Festival; B: the National Celebration; C: important study hospital inspection; D: one inpatient building with 400 beds was reconstructed.

**Figure 5:**
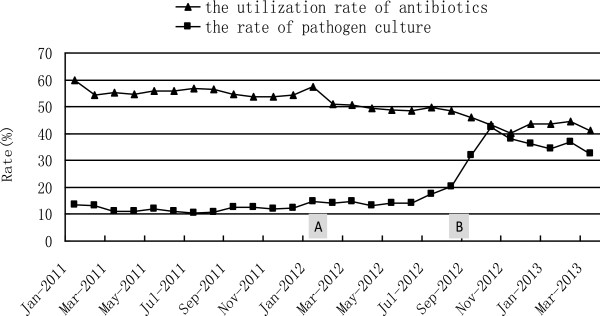
**Monthly utilization rate of antibiotics and the rate of pathogen culture in the hospital.** The monthly utilization rate of antibiotics was stable at about 55%; the rate of pathogen culture was stable at about 10% before Jan 2012 (A intervention). After A intervention, the former decreased to 50% and the latter rose to about 15%. When strict intervention B was implemented, the two rates were kept stable, at about 40% and 30% respectively. A intervention: monthly utilization rate of antibiotics and rate of pathogen culture in each ward were reported monthly and antibiotic public health education was provided to physicians. B intervention: in addition to A, the ICPs provided special education to different wards according to the ward’s condition; harsh economic measures were used on the wards with the worst results.

All the confirmed NIs were recorded by RT-NISS and the system automatically generated various data, and marked the status of the patient (e.g. admission/transfer/discharge). The data included (but was not limited to) NI rates per 100 inpatients, daily NI rates per 1,000 inpatients, the proportion of different infected sites, the distribution of pathogens, and antibiotic administration. The ICPs could easily obtain data at any time, and from any ward in the hospital, without any manual input. For example, Figure 4 illustrates the dynamic of daily NI rates across the hospital from 1^st^ Jan 2011 to 31^st^ Mar 2013. The monthly antibiotic use and microbiological culture results are shown in Figure 5.

The targeted surveillance module could calculate the targeted NI rate (i.e. SSI, CABSI). The system obtained the relative targeted surveillance data without the need for manually collecting such information. For example, all the operative inpatients’ AOS information (i.e. ASA score, blood loss, the time of operation), and prophylactic antibiotic administration times within 0.5 to 2 hours prior to their operation, could be automatically calculated.

## Discussion

RT-NISS is a real-time NI surveillance system that is useful for the wider hospital. Using our system, it takes 2 hours for one ICP to deal with 70 new suspicious NI alerts across 3,500 inpatients. ICPs only need several minutes per day to make decisions about suspicious outbreaks; the system also supports ICPs to have comprehensive knowledge about NIs risks across an entire hospital. ICPs can immediately undertake targeted professional measures to control NIs—just like shooting an arrow at a target. Furthermore, RT-NISS can help ICPs to target surveillance with greater efficiency, without the need for manually collecting large amounts of data. Most importantly, the daily surveillance data helped us greatly to improve our prevention and control measures. Long-term data might be used to further investigate the risk factors of NIs, which would benefit and inform targeted prevention and control programs.

Recently, many hospitals in the US, and approximately one third of California’s hospitals, have begun to use various automated surveillance systems. As a result, these hospitals may achieve more depth and breadth with implementing evidenced-based infection control practices in this part of the U.S [13].

Most studies [5-9] in this field are concerned with limited targeted NI surveillance, and real-time hospital-wide surveillance has been seldom reported. This is because there are two problems that are difficult to overcome. First, a computer algorithm for all NIs has been difficult to develop with the required sensitivity and specificity; it has to consider many screening conditions, and be able to deal not only with general wards, but also special wards. Second, even though there are computer algorithms with higher sensitivity and specificity, they have only been used for cross-sectional studies or retrospective reviews. So to realize a daily real-time prewarning capability, the system must retain all NI information and be able to undertake complicated time-serial alignments to identify and recognize new suspicious NIs on a daily basis.

Many studies in the past [14,15] have applied different NI screening computer algorithms (such as positive microbiology reports or instances of antibiotic administration) for retrospectively or prospectively detecting NIs. Unfortunately, the use of antibiotic administration has been shown to be sensitive but not very specific, because there were many cases of excessive antibiotic usage, which can lead to false positive findings, e.g. the prophylactic or empirical use of antibiotics. The criterion of a positive microbiology report has been shown to be more specific but easier to loose NIs, because the physicians do not always order microbiology cultures for NIs. Despite this, the above studies have provided plenty of basic data. Recently, some studies have attempted to combine different NI criteria to develop processes for automatic identification. *Brossette SE* et al. [16] reported a successful hospital-wide prospective system with good accuracy in 2006, which combined clinical microbiology (including serological and molecular testing) and patient status data; the sensitivity was 0.86 and specificity was 0.98. We suspect that a possible reason for the low sensitivity was that the computer algorithm did not consider antibiotic usage.

Learning from the merits of previous computer systems, our computer screening algorithm included positive microbiological examination, antibiotic administration, serological and molecular testing (e.g. C-reactive protein, calcitonin), imaging reports, fever, invasive device use, inpatients transfer data, and other variables. Additionally, some of the screening strategies were designed for special wards areas. Compared with the “gold standard” (manual survey of NIs), RT-NISS showed good sensitivity (98.8%) and specificity (93.0%). The sensitivity and specificity was calculated using all of the screening algorithms. Theoretically, it is better to obtain higher sensitivity during NI screening, and good specificity during NI confirmation. Algorithms need to improve specificity, but at the same time be balanced with good sensitivity. It is inevitable that any electronic screening surveillance system will report some false-positive results with relatively low specificity. A strength of our study was that it used RT-NISS to screen for suspicious NIs alerts, followed by the ICPs scrutiny of the alerts, which help to obtain the excellent levels of sensitivity and specificity. Using this method, the specificity increased from 93.0% to 99.3%, with a favorable kappa (0.93). However, when using the ICP’s diagnosis, the sensitivity decreased from 98.8% (84/85) to 94.1% (80/85) because four alerts that were confirmed via manual survey were excluded by the ICPs because the infection information provided by RT-NISS was insufficient for confirmation.

When RT-NISS was implemented in a hospital for the first time, or used in a cross-sectional study or retrospective review, the prewarning NI rate was at 10% for all inpatients. The NIs alerts number should vary with differing conditions in relation to the NI computer screening algorithm, such as the different rates of microbiological testing. In our study, the prewarning NI rate was 15.0% (146/974) by RT-NISS in the survey in March 2013, but it was 8.8% (948/10,765) in July 2011. The main reason for the different percentages was the different microbiological testing rate (32.5% in March 2013 vs 10.4% in July 2011, Figure 5). A positive microbiological testing result was an important criterion in the computer screening algorithm, so a higher rate should bring about more NI alerts.

The other strength of our study was the criterion development and evaluation of daily real-time prewarning surveillance. Our innovation was that RT-NISS recorded all NI information and undertook time-serial alignments, so as to identify and prewarn new suspicious cases each day. Consequently, the daily rate of NI alerts was about 2%, which was less than the total rates in the two studies (15.0% vs 8.8%). This dramatically reduced the daily work burden of the ICPs. Because of this, it was possible for the ICPs to confirm all of the alerts during their routine work, which resulted in real-time surveillance. Furthermore, the ICPs were able to focus on other important issues, such as providing training to healthcare workers (e.g. about adequate hand hygiene), promoting evidence-based interventions about rational antibiotic administration, preventing or controlling clusters or outbreaks, and supervising and helping healthcare workers to improve their knowledge about transmission-based precautions. With the help of RT-NISS, this has led to an improved understanding and enhanced cooperation between healthcare workers and ICPs for preventing NIs.

Our study has several limitations. First, medical records were not included in the computer screening algorithm because many of the words in the records were based on the doctors’ personal preferences, and did not follow formal terminologies. Second, the comparison between RT-NISS and the manual survey was not conducted on all 3,500 inpatients in the study hospital because there were insufficient ICPs. Third, in order to balance good sensitivity with specificity, the RT-NISS could not prewarn and identify all of the NI inpatients. However, the NIs that were lost were rare, and usually associated with mild infections. Finally, the RT-NISS could only monitor NIs when the patient was in hospital, many NIs probably occurred when the inpatients were discharged and were not under surveillance. Only a small number of NIs were prewarned by RT-NISS when the patients were readmitted to the same hospital, and the NI had happened after discharge.

## Conclusion

In conclusion, after 4 years of system development and daily clinical practice, RT-NISS has confirmed its operational capabilities and demonstrated favourable efficiency. To date, more than 300 large hospitals in China have adopted the RT-NISS with good effect. We anticipate that NI rates will fall in these hospital, with the help of this system.

## Competing interests

The authors declare that they have no competing interests.

## Authors’ contributions

YX Liu, MM Du, JJ Suo and YB Xing designed and developed the RT-NISS framework. MM Du, BW Liu implemented and improved the system. R Huo and CP Chen worked on the computer program. MM Du, N Jia and YX Liu drafted the manuscript. All of the authors read and approved the manuscript.

## Pre-publication history

The pre-publication history for this paper can be accessed here:

http://www.biomedcentral.com/1472-6947/14/9/prepub
